# Isozyme-Specific Ligands for *O*-acetylserine sulfhydrylase, a Novel Antibiotic Target

**DOI:** 10.1371/journal.pone.0077558

**Published:** 2013-10-22

**Authors:** Francesca Spyrakis, Ratna Singh, Pietro Cozzini, Barbara Campanini, Enea Salsi, Paolo Felici, Samanta Raboni, Paolo Benedetti, Gabriele Cruciani, Glen E. Kellogg, Paul F. Cook, Andrea Mozzarelli

**Affiliations:** 1 Department of Food Sciences, University of Parma, Parma, Italy; 2 Department of Pharmacy, University of Parma, Parma, Italy; 3 National Institute of Biostructures and Biosystems, Rome, Italy; 4 Molecular Discovery Limited, London, United Kingdom; 5 Department of Chemistry, University of Perugia, Perugia, Italy; 6 Department of Medicinal Chemistry and Institute for Structural Biology and Drug Discovery, Virginia Commonwealth University, Richmond, Virginia, United States of America; 7 Department of Biochemistry, University of Oklahoma, Norman, Oklahoma, United States of America; Institute of Enzymology of the Hungarian Academy of Science, Hungary

## Abstract

The last step of cysteine biosynthesis in bacteria and plants is catalyzed by *O*-acetylserine sulfhydrylase. In bacteria, two isozymes, *O*-acetylserine sulfhydrylase-A and *O*-acetylserine sulfhydrylase-B, have been identified that share similar binding sites, although the respective specific functions are still debated. *O*-acetylserine sulfhydrylase plays a key role in the adaptation of bacteria to the host environment, in the defense mechanisms to oxidative stress and in antibiotic resistance. Because mammals synthesize cysteine from methionine and lack *O*-acetylserine sulfhydrylase, the enzyme is a potential target for antimicrobials. With this aim, we first identified potential inhibitors of the two isozymes via a ligand- and structure-based *in silico* screening of a subset of the ZINC library using FLAP. The binding affinities of the most promising candidates were measured *in vitro* on purified *O*-acetylserine sulfhydrylase-A and *O*-acetylserine sulfhydrylase-B from *Salmonella typhimurium* by a direct method that exploits the change in the cofactor fluorescence. Two molecules were identified with dissociation constants of 3.7 and 33 µM for *O*-acetylserine sulfhydrylase-A and *O*-acetylserine sulfhydrylase-B, respectively. Because GRID analysis of the two isoenzymes indicates the presence of a few common pharmacophoric features, cross binding titrations were carried out. It was found that the best binder for *O*-acetylserine sulfhydrylase-B exhibits a dissociation constant of 29 µM for *O*-acetylserine sulfhydrylase-A, thus displaying a limited selectivity, whereas the best binder for *O*-acetylserine sulfhydrylase-A exhibits a dissociation constant of 50 µM for *O*-acetylserine sulfhydrylase-B and is thus 8-fold selective towards the former isozyme. Therefore, isoform-specific and isoform-independent ligands allow to either selectively target the isozyme that predominantly supports bacteria during infection and long-term survival or to completely block bacterial cysteine biosynthesis.

## Introduction

**Figure 1:**
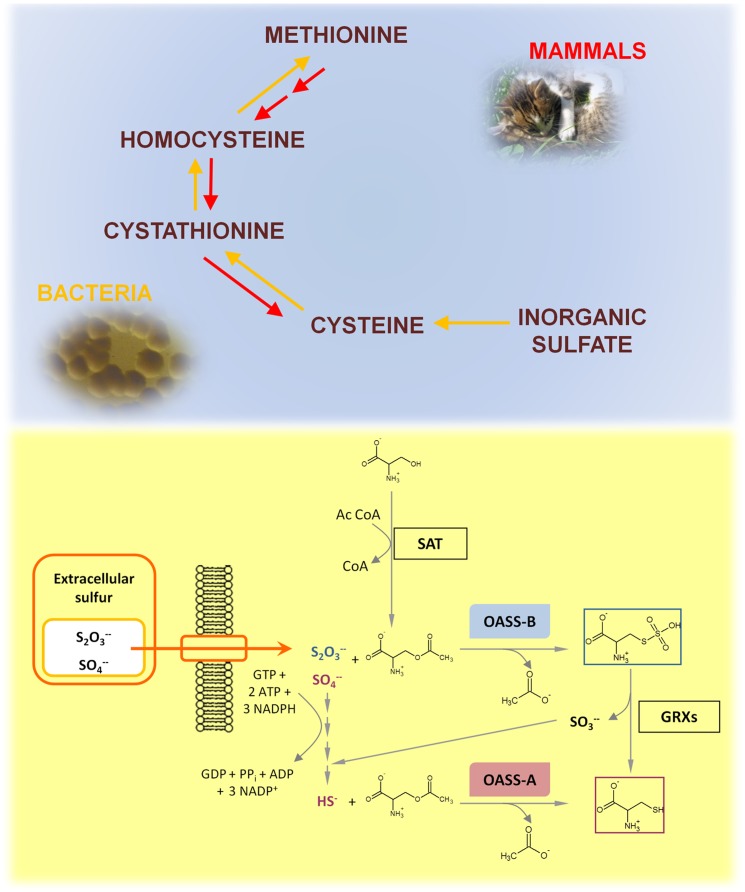
Cysteine biosynthesis. **Upper panel**: Intermediates of cysteine biosynthesis in mammals and bacteria. The red arrows indicate the biosynthetic pathway in mammals and the yellow arrows the biosynthetic pathway in bacteria. **Lower panel**: Sulfur assimilation in bacteria. Sulfate and thiosulfate are the most abundant forms of extracellular sulfur, the latter being predominant under less oxidizing conditions. Inorganic sulfur enters the cells through specific transporters. In contrast to OASS-A, OASS-B can directly use thiosulfate for cysteine biosynthesis. The product S-sulfo-L-cysteine is reduced by glutaredoxins to cysteine and sulfide that enters in the last step of the sulfate reduction pathway [120], [121].

In bacteria and plants cysteine is the only source of sulfur that is required for the synthesis of a variety of biomolecules, including methionine, Fe-S clusters, thiamine, glutathione, and biotin [1], [2]. In microorganisms, cysteine supplies the reducing power for protection against oxidative stress, either directly [3] or indirectly via reducing systems like glutathione/glutathione reductase, mycothione/mycothione reductase [4] or trypanothione/trypanotathione reductase [5]. In bacteria, cysteine is synthesized via the reductive sulfate assimilation pathway involving five enzymes (Figure 1). The cysteine regulon of pathogenic microorganisms is up-regulated, *in vitro*, under oxidative stress [6], in the presence of nitric oxide [7], and *in vivo*, during infection or long term survival [8], [9]. It has been proposed and experimentally proved that enzymes involved in sulfur metabolism, and specifically in cysteine biosynthesis, are targets for the development of novel antibiotics [4], [6], [10]–[23]. For example, *S. typhimurium* knock-out for cysteine synthase showed an increased susceptibility to ciprofloxacin, with a MIC 500-fold lower than wild type bacterium [24]. The mechanism of action of antimonials in the treatment of Leishmaniasis has been demonstrated to be linked to the biosynthesis of trypanothione and to a marked decrease in cellular thiol redox potential [25]. Furthermore, inactivation of enzymes involved in cysteine and methionine biosynthesis in *Mycobacterium tuberculosis*, significantly reduces bacterial virulence and persistence during the chronic phase of infection in mice [22]. Therapeutic strategies against microbes that rely heavily on sulphur metabolism for efficient host infection and colonization, such as *M. tuberculosis* and *Entamoeba histolytica,* have been proposed [4], [11].

In most bacteria and plants, cysteine biosynthesis culminates with the β-replacement of an activated serine derivative, *O*-acetylserine, by bisulfide, catalyzed by a family of enzymes known as *O*-acetylserine sulfhydrylases (OASS) [26]. OASS is a member of the cysteine synthase superfamiliy [26] and is a pyridoxal 5′-phosphate-dependent enzyme. Two OASS isozymes, OASS-A and OASS-B, have been identified that are differentially expressed depending on growth conditions. OASS-A is present at basal levels and is favored under aerobic conditions and in rich media, whereas OASS-B is expressed under anaerobic conditions [27]. The catalytic mechanism [27], [28], spectroscopic properties [29]–[32], and stability [33]–[35] of OASS-A have been characterized and compared with those of OASS-B [36]. The enzyme belongs to the fold type II of the PLP-dependent enzyme family [37], whose prototype is tryptophan synthase [38], [39]. The three-dimensional structure of OASS from different species was determined, including *Haemophilus influenzae, Escherichia coli, S. typhimurium, E. histolitica, Aeropyrum pernix, Thermotoga maritima, M. tuberculosis, Leishmania major* and *Arabidopsis thaliana*, either in the absence or presence of ligands [16], [36], [40]–[54].

The interaction of OASS with serine acetyltransferase (SAT), the preceding enzyme in the cysteine biosynthetic pathway, has been characterized determining both the binding affinity and kinetic mechanism [16], [55]–[63]. OASS-A forms a tight complex with SAT with a K_d_ in the nanomolar range [55], [61], whereas OASS-B does not interact with SAT [55], [56]. SAT binds to the OASS-A active site via its C-terminal peptide, resulting in a competitive inhibition of OASS [45], [61]. OASS-A forms a fast encounter complex with SAT, followed by a slow conformational change [64]. The structure of OASS-A from *H. influenzae* was determined with the C-terminal decapeptide of SAT bound in the active site [45]. Only the last four amino acids (NLNI) were detected, suggesting that they have a specific role in the energetics of the interaction. This conclusion is supported by extensive mutational and computational analysis [16], [61], also showing the relevance of the C-terminal amino acid isoleucine for OASS-SAT formation [51], [65]. The contribution of individual amino acids contained in the C-terminal sequence of SAT to complex formation and to binding specificity towards OASS-A and OASS-B was investigated using a small library of pentapeptides [19], [66]. Furthermore, recently, inhibitors for OASS-A have been obtained via a classical medicinal chemistry approach [18] and by virtual screening [11], [67].

For the identification of ligands specific for either OASS-A or OASS-B from *S. thyphimurium* we carried out complementary *in silico* and *in vitro* investigations. Our approach is based on the *in silico* screening of a subset of the ZINC library [68] with FLAP [69], docking with GOLD [70], [71] and re-scoring using HINT [72]. Many different approaches are exploited for *in silico* screening. As stated by Ma et al. [73] structure- or ligand-based virtual screening methods, usually based on fingerprinting, are used for simulating the interactions of a biomolecular target with compounds libraries in a rapid and cost-effective manner. FLAP belongs to this category of fingerprint methods, normally classified according to their dimensionality ranging from 1D to 3D [74]. The main strength of these approaches lies in their ability of comparing multiple fingerprints, i.e. a mathematical representation of a molecule, and computing their similarity using similarity coefficients [75]. Given the increased availability of computer power, docking approaches have been also exploited for screening and for investigating the binding mode of small molecules into the target binding pocket. Docking methods that have been developed and successfully applied in virtual screening experiments include AutoDock and AutoDock Vina [76]–[78]; DOCK [79]–[81]; FlexX [82]; Glide [83]; GOLD [84], [85]; Surflex [86], [87]. Their strengths and weaknesses, along with applications, have been reported by Bielska et al. [75]. Our choice of using FLAP was based on the available computational resource and on the positive results previously obtained [88]–[97].

The binding affinities of the best hits were evaluated *in vitro* on purified OASS-A and OASS-B, exploiting the change of PLP fluorescence emission upon binding [30]–[32], [55]. Ligands that bind to either OASS-A or OASS-B with K_d_ of 4–34 µM were identified. As a somewhat serendipitous result, ligands that bind to both isoforms with K_d_s in the micromolar range were also found. This result is fully explained by a few common pharmacophoric features of the active site, in spite of the completely distinct ability of interaction with SAT.

## Materials and Methods

### Virtual Screening

OASS-A and OASS-B structures from *S. typhimurium* were retrieved from the PDB database (PDB codes 1OAS [47] and 2JC3 [36], respectively). Structures were checked for chemically consistent atom and bond type assignments using the molecular modeling program Sybyl 8.1 (www.tripos.com). Amino-terminal and carboxy-terminal groups were set as protonated and deprotonated, respectively. The PLP atoms were renamed according to the GRID library (grub.dat) to allow the program to properly recognize the cofactor. Hydrogen atoms were computationally added using Sybyl Biopolymer and Build/Edit menu tools and energy-minimized using the Powell algorithm, with a convergence gradient ≤ 0.5 kcal (mol Å)^−1^ and a maximum of 1500 cycles.

The Specs database (www.specs.net) was chosen as starting library for performing virtual screening simulations. This database is part of the ZINC archive [68] (www.zinc.docking.org) and, according to previous experiences [89], [93], [98], contains molecules with significant chemical and geometric diversity, good purity and availability. A set of about 300,000 compounds was downloaded and filtered according to their LogP values calculated by Moka [99]–[101]. In order to assure sufficient solubility, only molecules with LogP ≤ 1 were retained, amounting, in this experiment, to 11,937. The pharmacophoric analysis and the virtual screening were performed with FLAP (Fingerprints for Ligands and Proteins) software [69], developed and licensed by Molecular Discovery Ltd. (www.moldiscovery.com). FLAP is based on the Molecular Interaction Fields (MIFs) calculated by GRID [102], used to describe small molecules and protein structures in terms of 4-point pharmacophoric fingerprints. FLAP MIFs provide a very accurate and efficiently compressed description of 3D molecular features and interactions modeled on the base of GRID MIFs. The fingerprint makes the method extremely fast, and is used for pose prediction and GRID MIFs similarity calculation, thus allowing to evaluate the complementarities of the ligands to the receptor. The algorithm calculates the GRID-MIFs for the template molecule derived from ligands (Ligand-Based Virtual Screening, LBVS) or from the pharmacophoric image of the binding site (Structure-Based Virtual Screening, SBVS) and for the screened compounds. The hotspots are combined in quadruplets (the-4 points). The quadruplets of each molecule contained in the database are compared with the quadruplets of the template. Matching quadruplets are used to overlay the compounds 3D-structure onto the template and, as determined by overlapping of the MIFs, the similarity is assigned to generate a 3D pharmacophoric hypothesis [69]. MIF similarity scores can be referenced to the best alignment obtained with a single probe, when the product of two or more probes is used. The ligand orientations simultaneously represent the best MIF alignment for a given probe. The FLAP approach has been successfully applied in several virtual screening analyses [88]–[97]. This procedure allows the quick removal of molecules with a low probability of interacting with the target and, thereby, selects the most interesting candidates with chemical and structural complementarities with the receptor binding site and/or its known ligands.

Before starting any virtual screening analysis, the molecules were minimized with the “mizer” module. Once screening was completed, the compounds from SBVS and LBVS were ranked according to the Global Sum score of FLAP and the distance to the model (FLAP distance score) [103]. In the perspective of a consensus scoring approach, the most promising candidates were docked into the binding pocket of the respective targets with GOLD, version 3.1 (www.ccd.cam.ac.uk), and then rescored with HINT [72]. For each compound, 50 diverse poses were generated and analyzed. A radius of 15 Å was used to direct site location. A maximum number of 100,000 operations were performed for each docking search, on a population of 100 individuals with a selection pressure of 1.1. Operator weights for crossover, mutation, and migration were set to 95, 95 and 10, respectively. The number of islands and the niche were set to 5 and 2. No constraints were imposed. Polar hydrogen atoms in the binding pocket were optimized for hydrogen bonding during docking simulations. The default GOLDScore fitness was used as native scoring function [71]. HINT was then used as post-docking processor scoring function [104]–[108]. The HINT score provides a quantitative evaluation of ligand-protein interaction as a sum of all individual atom-atom interaction contributions that is proportional to the free energy of binding, as previously described [16], [104].

All calculations were run on a 8 CPU workstation Intel(R) Xeon(R) CPU X5560 @ 2.80 GHz, with a 16 GB 1333 MHz RAM, and a Linux operating system RHEL 5.4 x86_64, kernel version 2.6.18–164.el5. Given the available computational resource, and good results previously obtained, the FLAP software represented to us the best choice for performing rapid and profitable virtual screening analysis. The application of other methods, such as docking, might result in the identification of different compounds, given their powerful search in the conformational space [75].

### Chemicals

Chemicals, purchased from Sigma-Aldrich, were of the best available quality and used as received. Experiments, if not otherwise indicated, were carried out in 100 mM Hepes buffer, pH 7.0, at 20 °C.

### Determination of ligand binding affinity to OASS-A and OASS-B

OASS-A and OASS-B were expressed and purified as previously described [19]. The binding affinity of selected ligands to OASS-A and OASS-B was determined by monitoring the increase in fluorescence emission of the bound PLP at 500 nm following excitation at 412 nm [16], [55]. Emission spectra were collected at increasing ligand concentrations in the presence of 0.05–1 µM OASS, 100 mM Hepes buffer, pH 7.0 at 20°C. DMSO and/or potassium hydroxide were added when needed to solubilize the compounds and pH was checked to be between 7 and 8.5. Spectra were corrected for the buffer contribution. Fluorescence measurements were carried out using a FluoroMax-3 fluorometer (HORIBA-Jobin Yvon), equipped with a thermostated cell-holder.

The dependence of fluorescence intensity at 500 nm on ligand concentration was fitted to a binding isotherm:
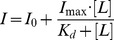
(1)where *I* is the fluorescence intensity at 500 nm in the presence of the ligand, *I_0_* is the fluorescence intensity in the absence of ligand, *I_max_* is the maximum fluorescence change at saturating ligand concentrations, [L] is the ligand concentration, and *K_d_* is the dissociation constant of the OASS-ligand complex. This measurement is a direct determination of ligand dissociation constant in the absence of substrate, thus it coincides with K_i_, independently from the inhibition mechanism.

## Results and Discussion

**Figure 2:**
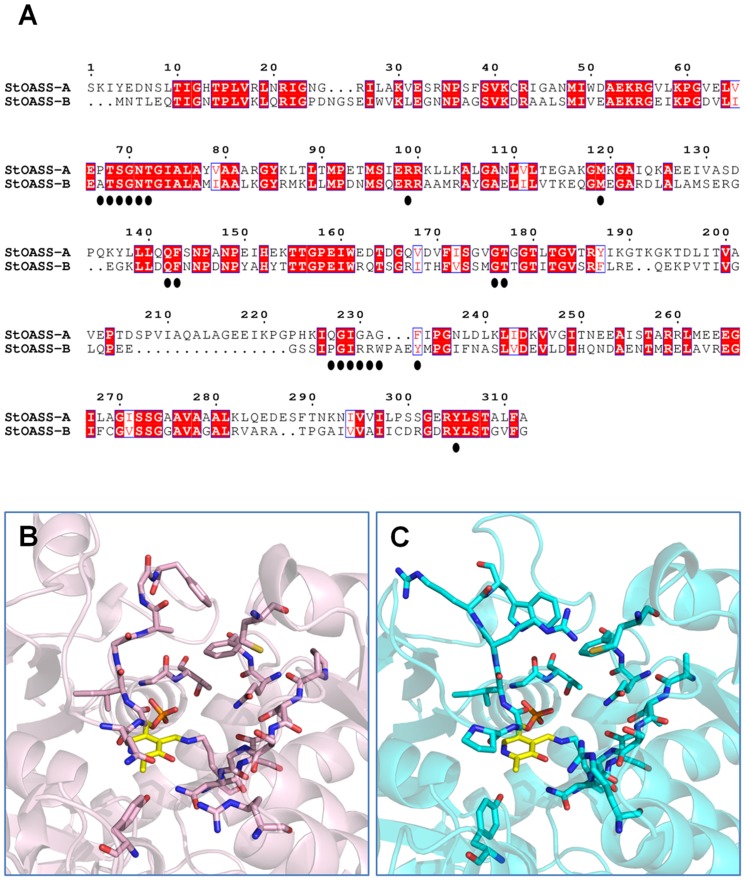
Structural comparison of OASS-A and OASS-B. **Panel A**: Structure-based amino acid sequence alignment of OASS-A and OASS-B from *Salmonella typhimurium*. The alignment, carried out on the PDB entries 1OAS and 2JC3 using the Flexible structure AlignmenT (FATCAT) method [122], gave an overall identity of 40.32% and a similarity of 56.51%. Identical residues have a red background and residues with similar physicochemical properties are shown in red. Similarity scores were calculated by the ESPript program [123] using the Blosum62 matrix set at global score of 0.2. Residues of the first active site shell are indicated by dark circles below the alignment. **Panel B**: Active site of OASS-A. Residues of the first active site shell and PLP are shown in ball and stick style, colored pink and yellow, respectively. **Panel C**: Active site of OASS-B. Residues of the first active site shell and PLP are shown in ball and stick style, colored cyan and yellow, respectively.

The biochemical investigation of OASS-A and OASS-B reactivity [36] and active site specificity probed by pentapeptides [19] indicate that, despite an overall 40% sequence identity and a 70% sequence identity for the first active site shell (Figure 2A), the two isozymes exhibit subtle but significant structural differences (Figure 2B,C). Most of the residues of the first active site shell are conserved, with residues belonging to the N-terminal domain (residues 1–12 and 35–145, OASS-A numbering [47]) showing a 90% identity, residue P67 being substituted by A69 in OASS-B. The larger divergence is observed in the loop around G228 in the front of PLP that has been suggested to undergo minor conformational changes during catalysis [47]. In particular, substitution Q227→P207 leaves one side of the pocket more accessible in OASS-B. In addition, as already discussed [19], G230 is substituted by R210, a residue that in some microorganisms plays a role in the selection of *O*-phosphoserine as the preferred substrate of the B isoform [53], [109]. The higher conservation degree of the residues belonging to the N-terminal domain with respect to those of the C-terminal domain allow the last two residues of pentapeptides docked in the active sites of OASS-A and OASS-B to occupy similar positions [19]. In spite of these common features, only OASS-A is able to interact with high affinity with SAT [55], [56].

### Ligand-Based Virtual Screening on OASS-A

**Figure 3:**
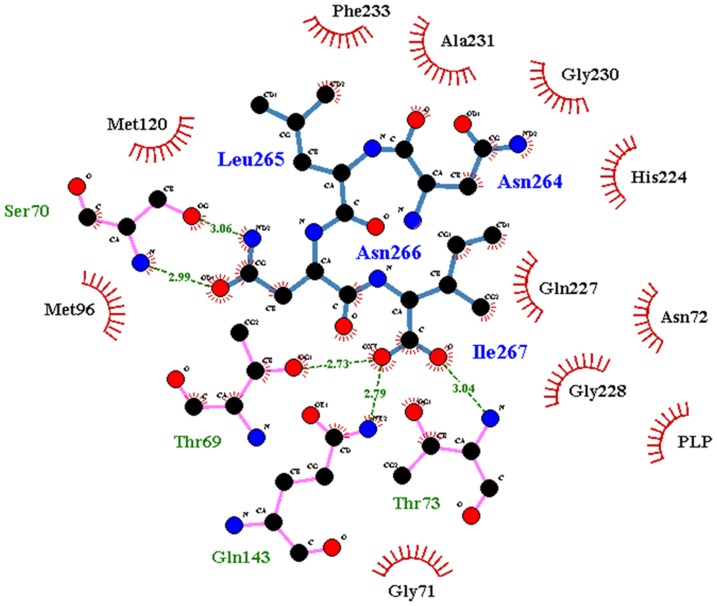
LigPlot of the wild type tetrapeptide ligand in the active site of *Haemophilus influenzae* OASS. The interactions between the Asn-Leu-Asn-Ile tetrapeptide and the active site residues of *H. influenzae* OASS-A (PDB code: 1Y7L) are reported. The figure was drawn with LigPlot program version 4.5.3 [124].

The structures of OASS-SAT or OASS-C-terminal peptide complexes from *S. typhimurium* are not available. Therefore, the LBVS was performed using the crystallographic structure of the two last residues of SAT, i.e. Asn266 and Ile267, complexed with OASS-A from *H. influenzae* (PDB code 1Y7L [45]) as a template. This choice is justified by the relatively high sequence (70%) and structural identity [110] between OASS-A from *H. influenzae* and from *S. typhimurium*, and the comparable affinity of the two enzymes for the *H. influenzae* SAT C-terminal pentapeptide (MNLNI), 44 µM and 120 µM, respectively [16], [19]. In particular, the main binding contribution is provided by hydrogen bonds formed between the peptide Ile carboxylate group and Thr69 and Thr73 (Thr68 and Thr72 in *S. typhimurium*), and by hydrophobic contacts between the PLP cofactor and Phe144 with the Ile side chain. (Figure 3). Asn at peptide position P4 is hydrogen bonded with Ser70 (Ser69 in *S. typhimurium*) and a water molecule and contributes more than 15% of the total interaction energy [16]. Analyses of the docking models of several pentapeptides and of some of the corresponding crystallographic structures indicate that a good OASS-A binder contains two hydrogen bond acceptor groups, i.e., the side chain of Asn and the carboxylate of Ile, and a hydrophobic moiety, i.e., the Ile side chain [16].

**Figure 4:**
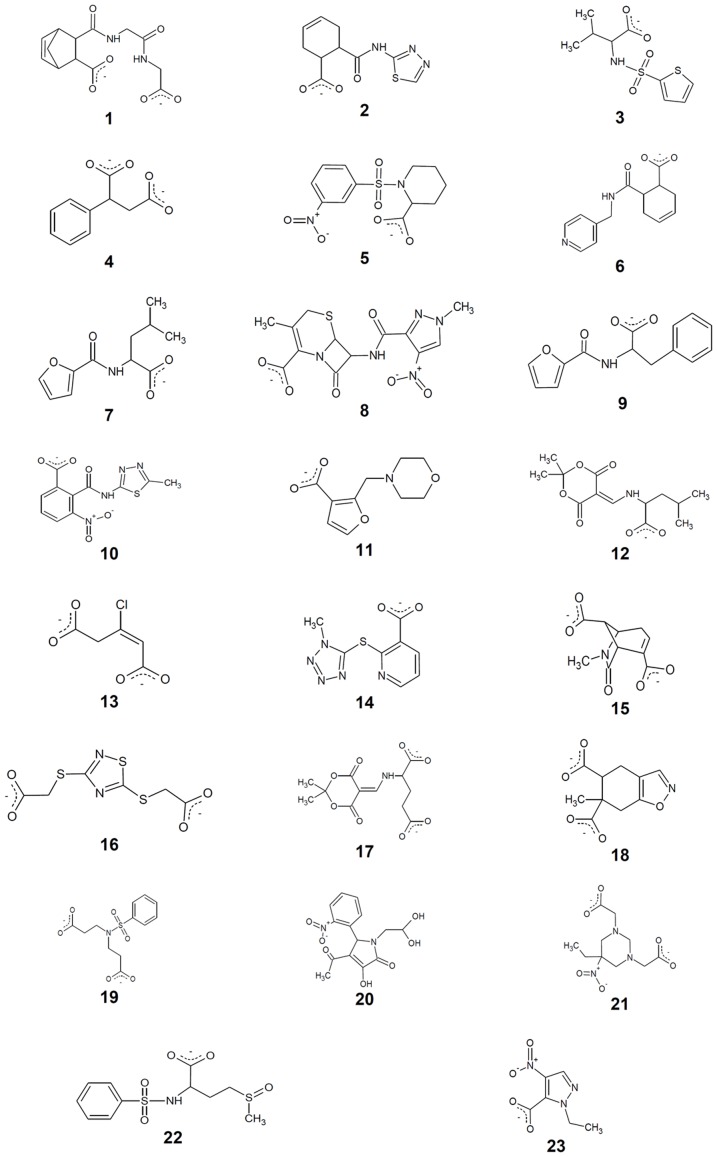
Compounds selected by SBVS/LBVS-docking procedures for OASS-A and OASS-B.

**Figure 5:**
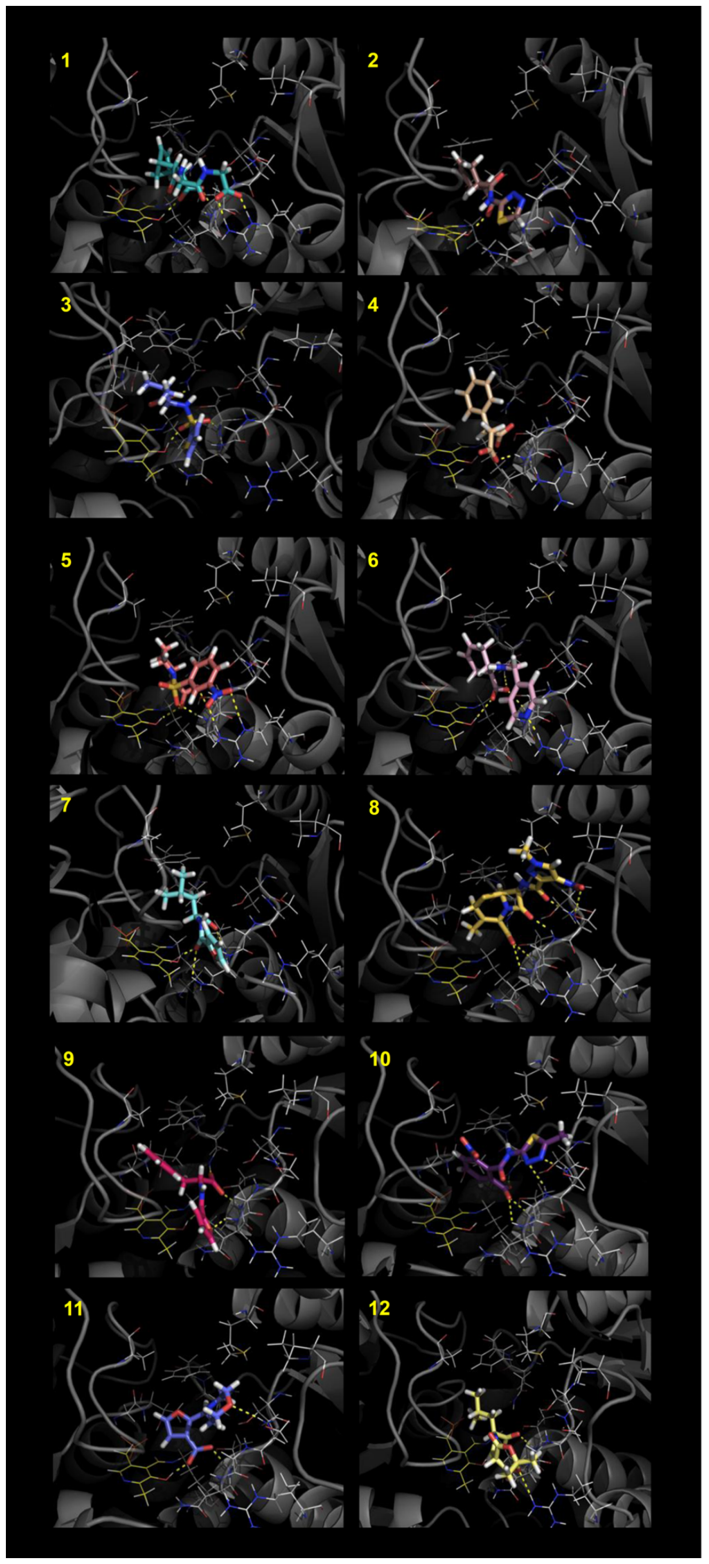
Best HINT scored conformations of the compounds selected by the SBVS/LBVS-docking procedures for OASS-A. The images were prepared with PyMOL (The PyMOL Molecular Graphics System, Version 1.5.0.4 Schrödinger, LLC.)

The LBVS set was initially composed of 1200 molecules showing Global Sum scores higher than 1.5. After individual inspection, molecules with at least one hydrogen bond acceptor group and a hydrophobic moiety were selected, docked into the binding site of OASS-A with GOLD, rescored with the HINT force field and again individually inspected. A HINT score value of 3000 was chosen as threshold, indicative of an energetically stable complex [111]. On the basis of: i) the generated conformations, ii) the interactions with the surrounding residues and iii) the HINT score value, seven compounds were selected for purchase and assays (Table 1 and Figures 4–5).

**Table 1:** List of compounds selected from virtual screening and tested against OASS-A.

Compound	Specs code	K_d_ (µM)
1	AO-623/14653116	3.7 ± 0.4
2	AK-968/12383180	82 ± 18
3	AQ-390/43356434	95 ± 10
4	AD-232/25000151	103 ± 9
5	AG-690/36829059	218 ± 61
6	AK-968/15253078	283 ± 19
7	AG-690/11214033	558 ± 131
8	AK-968/41922818	732 ± 72
9	AG-690/34035030	1300 ± 400
10	AG-690/11665608	> 1500
11	AG-664/25040003	> 1500
12	AP-060/40977348	> 1500

### Structure-based Virtual Screening on OASS-A

The structure-based analysis was performed using the OASS-A structure to generate the template [47]. On the basis of the Global Sum score, 600 compounds composed the initial SBVS set. These were inspected and analyzed as previously described. Following the pharmacophore hypothesis reported for OASS-A [16], compounds with two hydrogen bond acceptor groups were preferentially chosen, docked and re-scored with the HINT algorithm. The five molecules exhibiting higher scores and better pharmacophore profiles were selected for purchase (Table 1 and Figure 4–5) and assays.

### Structure-based Virtual Screening on OASS-B

**Figure 6:**
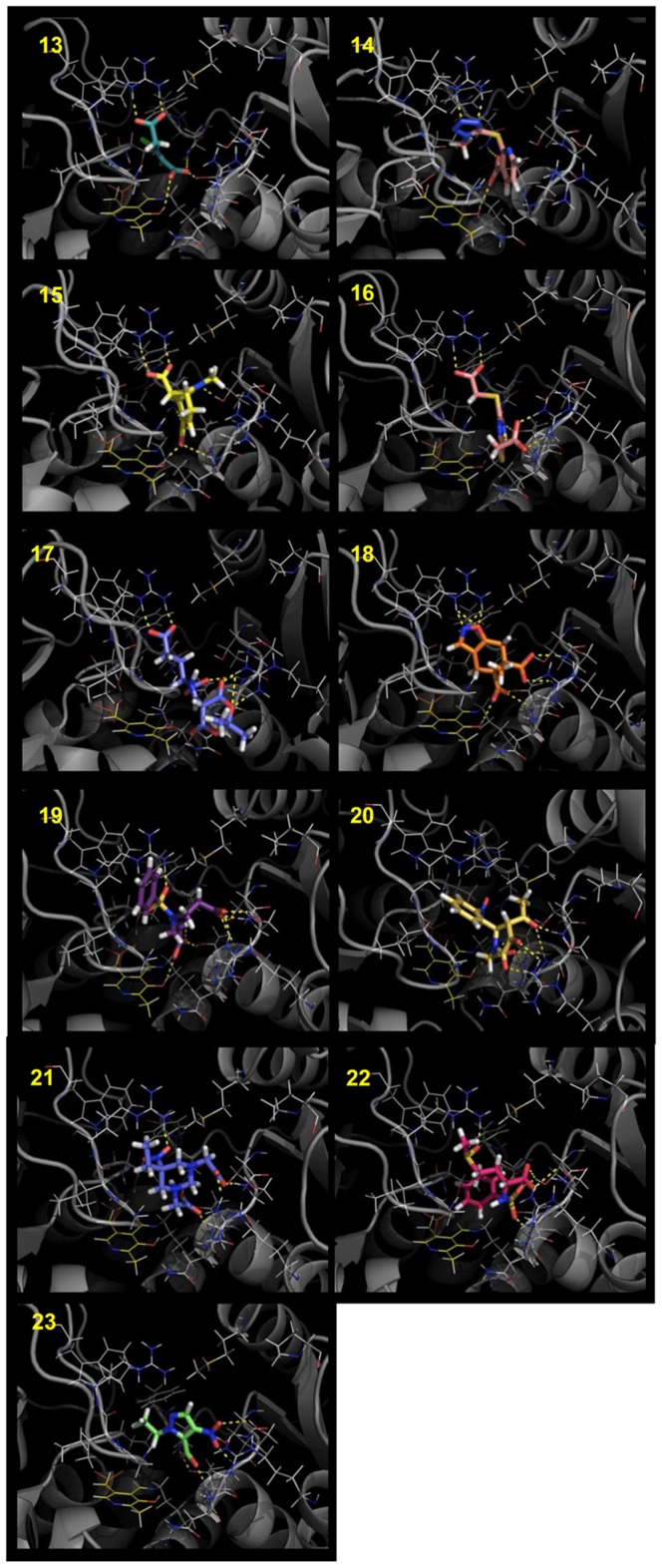
Best HINT scored conformations of the compounds selected by the LBVS/docking procedures for OASS-B. The images were prepared with PyMOL (The PyMOL Molecular Graphics System, Version 1.5.0.4 Schrödinger, LLC.)

A structure-based analysis was also performed to identify potential ligands of OASS-B, with a template based on the OASS-B crystal structure [36]. Eleven compounds were identified as potential binders (Table 2). The chemical structure of the selected molecules and their orientations in the active site of OASS-B are reported in Figure 4 and Figure 6. Since the SAT C-terminal peptide does not bind to OASS-B [55], [56], and no other OASS-B ligands are known to date, the LBVS approach was not performed for OASS-B.

**Table 2:** List of compounds selected from virtual screening and tested against OASS-B.

Compound	Specs code	K_d_ (µM)
13	AK-564/25068019	33±2
14	AI-204/34859016	810±110
15	AP-402/41884919	>1500
16	AG-664/25098006	>1500
17	AH-262/34614012	>1500
18	AC-776/15493018	>1500
19	AE-848/08323031	>1500
20*	AG-690/12134163	n.d.
21	AG-205/34690008	>1500
22	AO-080/42837941	> 1500
23	AK-968/41172226	>1500

* due to the strong emission at 500 nm for excitation at 412 nm, this compound was assayed at concentrations lower than 100 µM and no binding was observed.

### Selected OASS-A and OASS-B ligands

The twenty-three compounds selected as above described are characterized by at least a hydrogen acceptor group, i.e., a carboxylic group able to bind into the groove containing Asn71 and Thr72 (OASS-A numbering), and a hydrophobic moiety occupying the hydrophobic cleft lined by PLP, Phe143 and Thr177. Most of them also contain a second hydrogen acceptor group oriented towards Ser69 or Arg99. These features are consistent with the chemical properties of SAT peptides previously analyzed [16]. The identified molecules exhibit a molecular weight of 160-320 Da and most possess an aromatic moiety, except for compounds **1**, **12**, **13**, **15**, **17** and **21**. Many compounds in the set have one or two carboxylate groups.

Recently, virtual screening using the natural compounds subset of the ZINC database identified ten inhibitors of *E. histolytica* OASS, two of which possess dissociation constants in the micromolar and submicromolar range [11]. These inhibitor molecules are glycosides (gossypin and vitexin), aromatic compounds (pyrrole and pyrimidine derivatives) or polyhydroxylated compounds. Surprisingly, only three of them contain a carboxylate group and, of these, only one was found to be a good binder to OASS. Furthermore, the carboxylate group of these compounds is, in the reported models, surprisingly bound to a different site from that occupied by the carboxylate of the amino acid substrate [52]. The second best ligand is proposed to make hydrogen bonds with residues of the substrate-binding loop via a ketone carbonyl group. Similarly, virtual high-throughput screening on *M. tuberculosis* OASS-A led to the identification of fluoro- and nitro-substituted aromatic compounds [67]. The higher affinity compound from this search places a trifluoromethyl substituent in the binding pocket where the carboxylate of the amino acid substrate is bound. This compound was shown to be effective in inhibiting *M. tuberculosis* growth with a MIC of 7.6 µM. Very recently, structure-based and rational design approaches have led to the optimization of a thiazolidine inhibitor of *M. tuberculosis* OASS-A with IC_50_ in the nanomolar range [112].

### Determination of the dissociation constant of selected ligands towards OASS-A and OASS-B

A total of 12 compounds for OASS-A (Table 1) and 11 compounds for OASS-B (Table 2) predicted to be potential ligands were experimentally tested by determining their dissociation constants exploiting PLP fluorescence changes as a function of ligand concentration [55]. The observed increases in coenzyme fluorescence and the concomitant blue shifts of the peaks upon ligand binding result from the closure of the active site – thus altering the coenzyme microenvironment [29], [55]. It is important to point out that the evaluated dissociation constants for these compounds correspond to their inhibition constants, K_i_, because they occupy the enzyme active site [18], [19], and, therefore, are purely competitive inhibitors [113].

**Figure 7:**
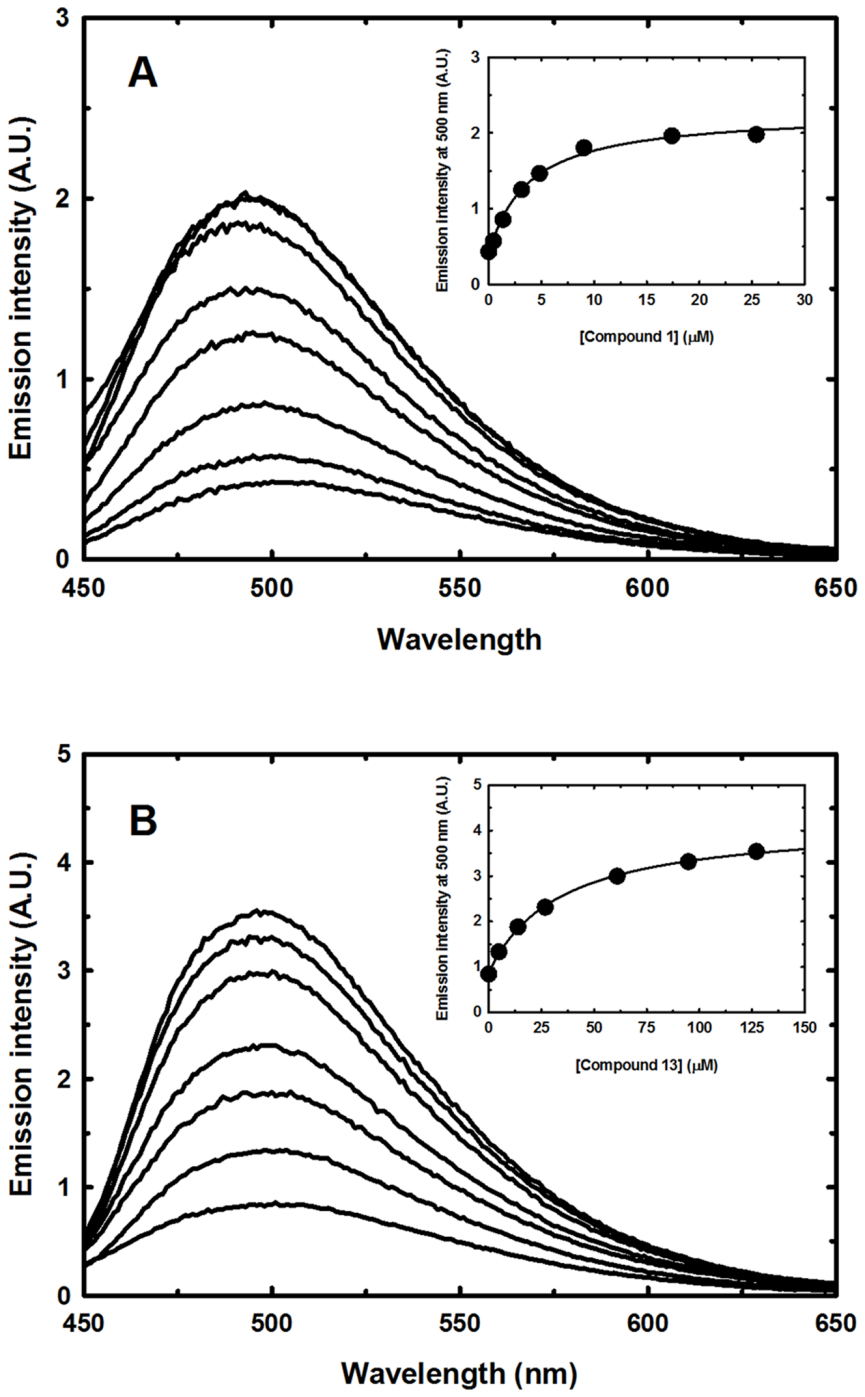
Binding of ligands to StOASS. **Panel A**. Fluorescence emission spectra upon excitation at 412 nm (slit_ex_ =  6 nm, slit_em_ =  6 nm) of a solution containing 50 nM StOASS-A and increasing concentrations of Compound **1** in 100 mM Hepes buffer, pH 7.0, at 20°C. Inset: Dependence of the fluorescence emission intensity at 500 nm on the ligand concentration. The line drawn through data points is the fit to a binding isotherm with K_d_  =  3.7 ± 0.4 µM. **Panel B**. Fluorescence emission spectra upon excitation at 412 nm (slit_ex_ =  4 nm, slit_em_ =  4 nm) of a solution containing 1 µM StOASS-B and increasing concentrations of Compound **13** in 100 mM Hepes buffer, pH 7.0, at 20 °C. Inset: Dependence of the fluorescence emission intensity at 500 nm on the ligand concentration. The line drawn through data points is the fit to a binding isotherm with K_d_  =  33 ± 2 µM.

**Figure 8:**
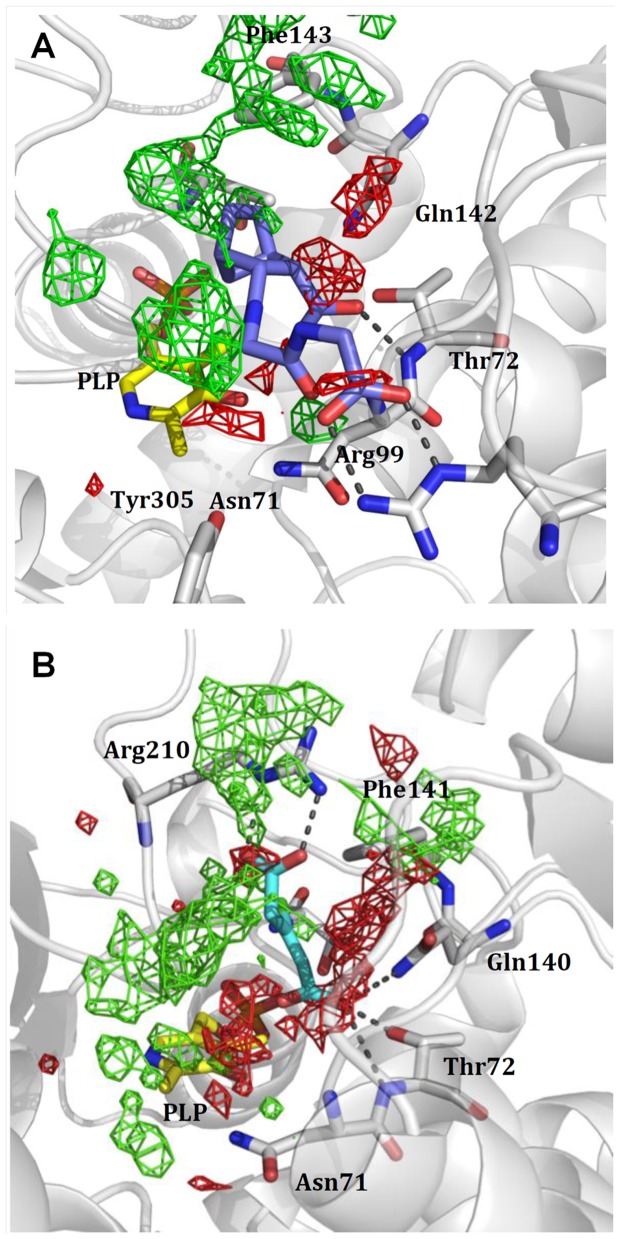
Docking pose of best binders to the two isozymes placed into the active sites. **Panel A**: Docking pose of **1** in the OASS-A binding pocket. Red and green contours identify the hydrogen bond acceptor and hydrophobic GRID MIFs. Hydrogen bond donor hot spots have not been shown for clarity. **Panel B**: Docking pose of compound **13** in the OASS-B binding pocket. Red and green contours identify the hydrogen bond acceptor and hydrophobic GRID MIFs. Hydrogen bond donor hot spots have not been shown for clarity.

Representative fluorimetric titrations at increasing concentrations of **1** are reported for OASS-A (Figure 7a) and at increasing concentrations of **13** for OASS-B (7b). Four compounds exhibited K_d_ for OASS-A equal to or lower than 100 µM, four between 100 µM and 1 mM, and four exhibited a K_d_ higher than 1.5 mM (Table 1). Compound **1** shows the lowest dissociation constant for OASS-A, 3.8 µM. Analysis of the docked model for **1** in the OASS-A active site predicts that the compound is well positioned in the pocket and is able to contact Thr72 with one carboxylic moiety, Arg99 with the second, and to fill the hydrophobic cleft with its byciclic moiety (Figure 5/1 and Figure 8a). The proper location of each moiety is also testified by the superposition with the corresponding GRID MIFs reported in Figure 8a, where both carboxylic groups lie in H-bond acceptor regions (red contours), and the byciclic moiety in a hydrophobic-favorable area (green contour). Interaction with Arg99 was not predicted for the wt peptide docked in StOASS-A active site, whereas contacts between Arg99 and the tyrosine at position P4 were predicted in the docked pose of MNYDI, the highest affinity peptide for StOASS-A (K_diss_ =  220 nM) [19]. Compound **2** still exhibits a reasonable affinity towards OASS-A, i.e., K_d_  =  82 µM, but lacks a stable salt bridge with Arg99 (Figure 5/2). Compound **3** shows a similar K_d_ (95 µM), and in spite of its not perfectly predicted localization in the binding pocket, it completely fits the pharmacophoric requirement, with a carboxylic and a sulfonamide group, as well as an isopropyl hydrophobic moiety (Figure 5/3). Models of Compound **4** predict that it places both one carboxylic moiety and a hydrophobic group in the good positions, but the short distance between the two carboxylic groups does not allow the second to reach Arg99 (Figure 5/4). Compounds **5** and **6** (Figures 5/5 and 5/6, respectively) are similarly predicted from models to be located in the binding pocket, contacting both Thr72 and Arg99, but the second H-bond acceptor moiety, i.e. a nitro group in 5 and a pyridine nitrogen in 6, appears to be less effective than that of **1**. A slightly different orientation is predicted for **7**, whose carboxylic moiety interacts with both Thr72 and Gln142, while the furan ring oxygen barely reaches Asn71 (Figure 5/7). Compounds **8** and **10** (Figure 5/8 and 5/10, respectively) – the latter showing significant less affinity towards the target – are modelled to occupy the binding site in a different orientation, as they interact with Asn71, Thr72 and Gln142, and with Ser69. Compound **9** (Figure 5/9) was selected from the virtual screening because of the presence of a carboxylic group and a nearby aromatic hydrophobic ring, and compound **11** was selected because our docking model suggests that it is properly located and able to contact both Thr72 and Ser69 (Figure 5/11). However, neither of them binds to OASS-A in our assay. Likewise, no binding was observed for **12** (Figure 5/12), probably due to the presence of the additional hydrophobic moiety that might prevent complex formation.

Of the 11 molecules predicted to bind to OASS-B (Table 2), two, **13** and **14**, exhibited K_d_s of 33 ± 5 µM and 810 ± 110 µM, respectively. Compound **13** is characterized by its small size, high hydrophobicity, and the presence of a chlorine that docking models (Figure 6/13 and Figure 8b) predict as properly located in the pocket hydrophobic region. The model also shows hydrogen bonds formed by the ligand carboxylate moieties with residues Thr272 and Gln140 on one side and Arg210 on the other. A similar interaction profile is also exhibited by compound **1**, the best OASS-A binder. While models of **13** show that it does not completely fill the large binding cavity of OASS-B, both hydrogen bond acceptor groups and the hydrophobic chlorine correspond to hot spots of the binding pocket, i.e., the red and green GRID contours, respectively (Figure 8b). Compound **13** is structurally similar to the cyclopropane-1-carboxylic acids derivatives identified by some of us [18] and displaying high affinity for the A isozyme from *H. influenzae* (HiOASS-A). This structure-activity relationship study showed that trans-2-substituted cyclopropane-1-carboxylic acids were better binders than cis-2-substituted molecules. Docking of (±)-*trans*-2-[(1*E*)-prop-1-en-1-yl]cyclopropanecarboxylic acid in the binding site of HiOASS-A revealed that the hydrophobic pocket of the enzyme was occupied by the propenyl moiety in a pose similar to the binding pose of **13**, with the chlorine substituent, also placed in trans configuration with respect to the carboxylic moiety, properly located in the pocket hydrophobic region. In agreement with the computational results, a significantly higher K_d_ value was measured for **14** (Figure 6/14), which is predicted to interact with Thr72 and Arg210 through its tetrazole ring, and places the hydrophobic nicotinic ring in front of Arg99. No binding was detected for any other of the selected molecules, i.e., **15**–**23** (Figure 6/15-6/23), possibly because of their bulkier substituents, or the absence of strong salt bridges with Arg210.

### Ligands for both OASS-A and OASS-B

**Figure 9:**
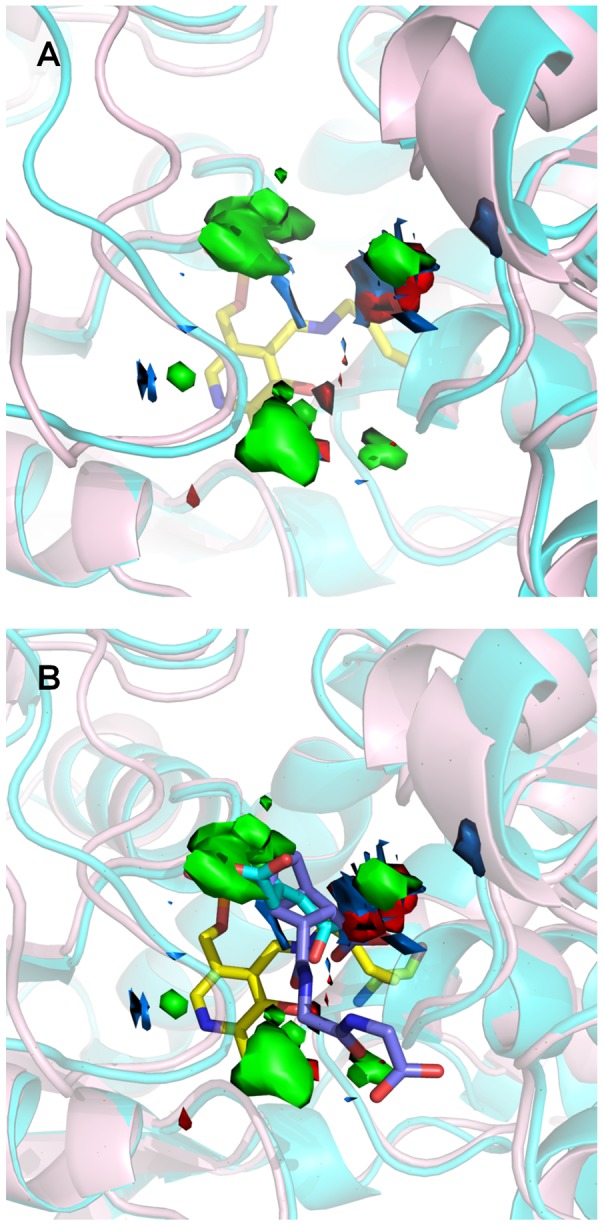
GRID MIFs calculated for OASS-A and OASS-B. Red, blue and green contours identify the hydrogen bond acceptor, hydrogen bond donor and hydrophobic MIFs, respectively, calculated for OASS-A (pink cartoons) towards OASS-B (cyan cartoons). In **Panel B** compounds **1** and **13** are shown in ball and stick.

The common MIFs generated by GRID for OASS-A and OASS-B are reported in Figure 9A. The similarity of the scaffold identified for the two OASS isozymes reflects the pharmacophoric similarity of the two binding sites. Hydrogen bond acceptor and donor MIFs (red and blue contours, respectively) are nearly conserved, with the exception of a small red contour in OASS-B placed over the PLP, just in front of Arg210 (Ala231 in OASS-A) (Fig 9A). This observation led us to test the compounds characterized by the lowest dissociation constants for OASS-A against OASS-B and vice versa (Table 3). The three compounds showing dissociation constants lower than 100 µM towards OASS-A, i.e., **1**, **2** and **3**, were tested against OASS-B. Only **1** was able to bind OASS-B, with a dissociation constant of 50 µM. Of the two compounds that bind to OASS-B, i.e., **13** and **14**, compound **13** binds to OASS-A with a dissociation constant of 29 µM, thus exhibiting similar affinity for both isozymes. These findings deserve notice, due to the previous observation that peptide ligands [19] always showed a higher affinity for the A isoform with respect to the B isoform. This was explained as a filter mechanism that evolved to prevent binding of SAT to OASS-B. The identification of a ligand specific for the A isoform (**1**) and a ligand that binds with good affinity to both isoforms (**13**) opens the way to the development of more potent inhibitors of cysteine biosynthesis in pathogenic bacteria. In particular, **13** has the properties of a good lead as it is completely unadorned and has accessible chemistry. In addition, although the presence of a vinyl halide moiety could in principle confer reactivity towards residues of the active site, similarly to the chemistry observed, e.g., for γ-vinyl-GABA [17], no reaction or time-dependent inactivation of OASS was observed under our experimental conditions.

**Table 3:** List of compounds tested against both OASS-A and OASS-B.

Compound	K_d_ OASS-A (µM)	K_d_ OASS-B (µM)
1	3.7 ± 0.4	50 ± 5
2	82 ± 18	> 1500
3	95 ± 10	> 1500
13	29 ± 3	33 ± 2
14	> 1500	810 ± 110

Our computational/experimental procedure has been quite successful in identifying OASS inhibitors from a relatively small library. However, the affinities of compound 1 and 13 are still in the micromole range. According with the orientation assumed by the ligands into the model provided by GOLD and, most of all, with the extension of the MIFs calculated by FLAP, modifications might be introduced in order to optimize compounds towards the corresponding target. For instance, in the case of compound 1, the bicyclo heptene moiety could be extended towards Met119, Phe143 and Ala231, and functionalized with a H-bond acceptor group for contacting Gln142 on one side or with a H-bond donor group on the other side for contacting the PLP phosphate group. Also in the case of compound 13, a H-bond donor group could be added to contact the PLP phosphate. Moreover, a more bulkier substituent bearing a H-bond acceptor moiety like a carboxylate, could be introduced on carbon 2, to reach Arg99 and form a salt bridge.

## Conclusions

The biological roles of OASS-A and OASS-B in *S. typhimurium* virulence and persistence in the host are still unclear despite the large number of studies dealing with their detailed biochemical and biological characterization [27], [28], [35], [47], [114]–[116]. The knowledge of the relative abundance and regulation by effectors of the two isozymes during infection is a relevant missing information that can contribute to the pharmacological exploitation of these targets. For example, it has been recently shown that the activity of OASS-B on thiosulfate could represent an energy saving path to cysteine biosynthesis and could be preferred in metabolic conditions where the conservation of ATP and NADPH is important [117]. In addition, very recently, works by Hayes and coworkers [118], [119], identified OASS as the activating factor for a toxin that controls contact-dependent growth inhibition in *E.coli*. Surprisingly, the interaction between OASS and the toxin exploits the same mechanism of SAT-OASS complex formation, e.g. insertion of the C-terminal peptide in the OASS active site. Hence, OASS is a multifaceted enzyme whose function may indirectly influence processes such as long-term survival inside the host and biofilm formation.
